# An updated meta-analysis of the association between fibroblast growth factor receptor 4 polymorphisms and susceptibility to cancer

**DOI:** 10.1042/BSR20192051

**Published:** 2020-10-23

**Authors:** Abdolkarim Moazeni-Roodi, Sahel Sarabandi, Shima Karami, Mohammad Hashemi, Saeid Ghavami

**Affiliations:** 1 Tropical and Communicable Diseases Research Centre, Iranshahr University of Medical Sciences, Iranshahr, Iran; 2 Department of Clinical Biochemistry, Iranshahr University of Medical Sciences, Iranshahr, Iran; 3 Department of Clinical Biochemistry, School of Medicine, Zahedan University of Medical Sciences, Zahedan, Iran; 4 Genetics of Non-Communicable Disease Research Center, Zahedan University of Medical Sciences, Zahedan, Iran; 5 Department of Human Anatomy and Cell Science, Max Rady College of Medicine, Rady Faculty of Health Sciences, University of Manitoba, Winnipeg, MB, Canada; 6 Research Institute in Oncology and Hematology, CancerCare Manitoba, University of Manitoba, Winnpeg, Canada

**Keywords:** Cancer, FGFR4, Meta-analysis, Polymorphism, Susceptibility

## Abstract

Fibroblast growth factor receptor 4 (FGFR4) is a cell surface receptor tyrosine kinases (RTKs) for FGFs.

Several studies have focused on the association between FGFR4 polymorphisms and cancer development. This meta-analysis aimed to estimate the association between *FGFR4* rs351855 (Gly^388^Arg), rs1966265 (Val^10^Ile), rs7708357, rs2011077, and rs376618 polymorphisms and cancer risk. Eligible studies were identified from electronic databases. All statistical analyses were achieved with the STATA 14.0 software. Pooled odds ratios (ORs) with 95% confidence intervals (CIs) were used to quantitatively estimate the association. Overall, no significant association was found among rs351855, rs2011077, and rs376618 polymorphisms with the risk of overall cancer. The rs1966265 polymorphism significantly decreased the risk of cancer in recessive (OR = 0.87, 95% CI = 0.78–0.97, *P*=0.009, TT vs CT+CC) genetic model. Whereas the rs7708357 polymorphism was positively associated with cancer risk in dominant (OR = 1.17, 95% CI = 1.02–1.36, *P*=0.028) genetic model. Stratified analysis revealed that rs351855 variant significantly increased the risk of prostate cancer in heterozygous (OR = 1.16, 95% CI = 1.02–1.32, *P*=0.025 AG vs GG), dominant (OR = 1.20, 95% CI = 1.06–1.35, *P*=0.004, AG+AA vs GG), and allele (OR = 1.22, 95% CI = 1.06–1.41, *P*=0.005, A vs G) genetic models.

In summary, the findings of this meta-analysis indicate that rs1966265, rs7708357, and rs351855 polymorphisms are correlated to cancer development. Further well-designed studies are necessary to draw more precise conclusions.

## Introduction

Cancer poses a major health problem in both developing and developed countries [1–3]. There were approximately 18.1 million new cases and 9.6 million cancer deaths in 2018 [4]. The exact mechanism of cancer development is not clear yet. Mounting evidence have indicated that cancer development and progression is influenced by environmental and genetic factors [3,5–7].

The human fibroblast growth factor receptors (FGFRs), a subfamily of cell surface receptor tyrosine kinases (RTKs), consist of four closely related family members (FGFR1–4) [8]. FGFR activation by a various fibroblast growth factors (FGFs) triggers a cascade that leads to the activation of multiple signal transduction pathways, including the Ras/Raf/MapK, PI3K/Akt, STAT, and PLCγ, which can promote cell survival, cell proliferation, tissue development, differentiation, angiogenesis, epithelial-to-mesenchymal transition (EMT), angiogenesis, and can thereby involve in carcinogenesis [9–11].

The human *FGFR4* gene, also termed as cluster of differentiation 334 (CD334), is mapped to chromosome 5 (5q 35.1) [12] and is highly polymorphic. A common nonsynonymous single nucleotide polymorphism (SNP) at codon 388 (rs351855 G>A) in exon 9, which results in a change of glycine to arginine (Gly^388^Arg), was recognized in the transmembrane domain of the EGFR4 receptor [13]. Several studies inspected the relationship between *FGFR4* gene rs351855 G>A polymorphism and numerous types of cancer including breast cancer [13–18], cervical cancer [19–21], colon cancer [13,18,22], gastric cancer [23], prostate cancer [24–27], head and neck squamous cell carcinoma (HNSCC) [28,29], oral squamous cell carcinoma (OSCC) [30,31], lung cancer [32–34], hepatocellular carcinoma [35–37], sarcoma [38], skin cancer [39], neuroblastoma [40], non-Hodgkin’s lymphoma [41], and glioma [42]. There are few direct reports about the effect of FGFR4 polymorphism on the gene expression. *FGFR4* rs351855 polymorphism induced higher expression of FGFR4 protein and worse prognosis in breast cancer [43]. It has been reported that the rate of degradation of the Arg^388^ receptor was slower than the Gly^388^ receptor in neuroblastoma cells and also initiated internalization of the receptor into multivesicular structures (Rev1-1) [40]. In another investigation, the researchers showed that expression of the FGFR4 Arg^388^ protein activated the extracellular signal-related kinase pathway with subsequent expression of several genes which were associated with the aggressive form of prostate cancer [44], (Rev1-1). Researchers have reported that there was not any significant difference between different genotypes of *FGFR4* in gastric cancer [45]. Interestingly in the lung normal tissue, genotype-dependent transcriptional profile is present [46]. In the past few years, there were few epidemiological analysis and meta-analysis focusing on *FGFR4* in uterine leiomyomata [47], hip bone geometry [48–50], and all types of cancer [31,51]. Our current meta-analysis covers Gly^388^Arg rs351855 G>A and Val^10^Ile rs1966265 polymorphism in *FGFR4* polymorphisms to cancer susceptibility and provide wider information in this important regulator of cancers (Rev 1-2).

## Methods

### Literature search and inclusion criteria

**Figure 1:**
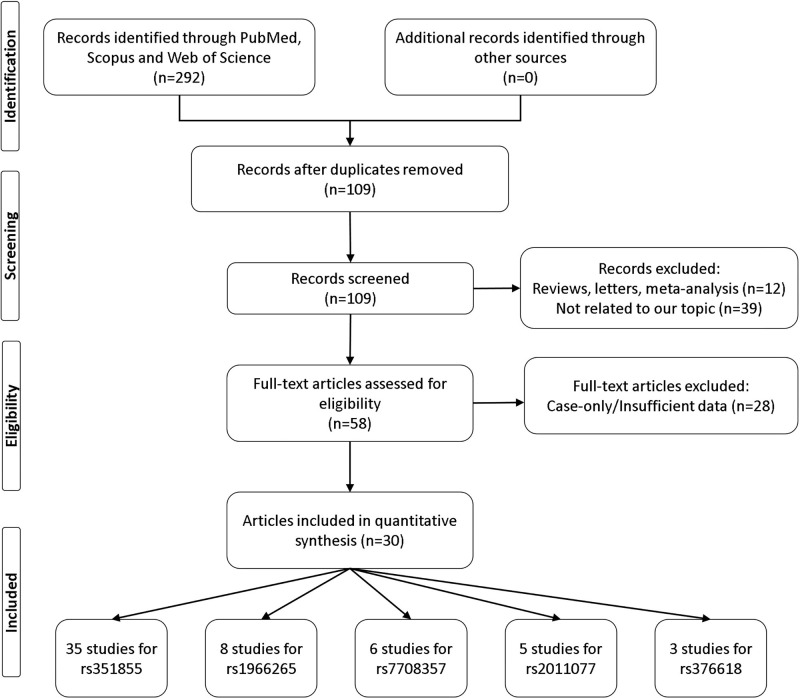
Flow chart illustrates the detailed study selection process of this meta-analysis

We performed a literature research for all eligible articles regarding the association between *FGFR4* polymorphisms on multiple electronic databases including Web of Science, PubMed, Scopus, and Google Scholar databases through using the following terms: ‘FGFR4 OR CD334’ AND ‘polymorphism OR, SNP, OR variation OR mutation’ AND ‘cancer OR carcinoma OR neoplasm OR tumor’ up to 10 May 2020. Besides, we also screened references of the included studies. Figure 1 shows the process of studies selection. Relevant studies included the meta-analysis if they met the following inclusion criteria: (1) original case–control studies addressing the correlation between *FGFR4* polymorphisms; (2) studies containing sufficient genotype data in both cases and controls; (3) the largest sample sizes were selected when repeatedly published articles by the same team. The exclusion criteria were: (1) conference abstract, case reports, reviews, duplication data; (2) insufficient genotype data provided.

### Data extraction

Two investigators independently screened the literature and extracted data from eligible studies according to exclusion and inclusion criteria. The following data were collected from each study including the first author’s name, publication year, country, ethnicity of participants, cancer type, genotyping methods, the sample size, and the genotype and allele frequencies of cases and controls (Table 1).

**Table 1:** Characteristics of the studies eligible for meta-analysis

First author	Year	Country	Ethnicity	Type of cancer	Source of control	Genotyping method	Case/control	Cases					Controls					HWE	Score
**Gly^388^Arg rs351855 G>A**								**GG**	**AG**	**AA**	**G**	**A**	**GG**	**AG**	**AA**	**G**	**A**		
Ansell	2009	Sweden	Caucasian	HNSCC	PB	PCR-RFLP	110/192	61	49		-	-	81	111		-	-	-	9
Bange	2002	Russia	Caucasian	Breast	PB	PCR-RFLP	61/123	26	28	7	80	42	55	60	8	170	76	0.114	7
Bange	2002	Germany	Caucasian	Breast	PB	PCR-RFLP	84/123	41	34	9	116	52	55	60	8	170	76	0.114	8
Bange	2002	Italy	Caucasian	Colon cancer	PB	PCR-RFLP	82/123	37	38	7	112	52	55	60	8	170	76	0.114	8
Batschauer	2011	Brazil	Caucasian	Breast	PB	PCR-RFLP	68/85	39	26	3	104	32	47	35	3	129	41	0.249	9
Chen	2018	Taiwan	Asian	Cervical cancer	HB	TaqMan	226/335	69	101	56	239	213	96	165	74	357	313	0.845	9
Chou	2017	Taiwan	Asian	OSCC	PB	TaqMan	955/1191	225	524	206	974	936	334	596	261	1264	1118	0.873	11
Fang	2013	China	Asian	NSCLC	HB	Sequencing	629/729	193	331	105	717	541	163	391	175	717	741	0.049	9
FitzGerald	2009	U.S.A.	Caucasian	Prostate	PB	SNPlex	1254/1251	587	544	123	1718	790	631	496	124	1758	744	0.070	15
FitzGerald	2009	U.S.A.	African	Prostate	PB	SNPlex	146/80	104	39	3	247	45	60	18	2	138	22	0.646	13
Gao	2014	China	Asian	NHL	NA	PCR-RFLP	421/486	117	189	115	423	419	171	240	75	582	390	0.541	11
Heinzle	2012	Austria	Caucasian	Colon cancer	PB	TaqMan	85/1660	42	33	10	117	53	802	723	135	2327	993	0.114	10
Ho	2009	Singapore	Asian	HCC	PB	Sequencing	58/88	27	17	14	71	45	30	38	20	98	78	0.241	6
Ho	2010	U.K.	Caucasian	Prostate	PB	TaqMan	397/291	183	182	32	548	246	150	117	24	417	165	0.860	11
Hosseini	2017	Iran	Asian	Breast Cancer	PB	PCR-RFLP	126/160	87	33	6	207	45	54	57	49	165	155	<0.001	6
Jiang	2015	China	Asian	Breast cancer	NA	Snapshot	747/716	205	404	138	814	680	270	348	98	888	544	0.398	12
Li	2017	China	Asian	Cervical Cancer	HB	PCR-RFLP	162/162	35	79	48	149	175	50	72	40	172	152	0.170	8
Ma	2008	Japan	Asian	Prostate	HB	PCR-RFLP	492/179	163	196	133	522	462	67	87	25	221	137	0.701	10
Mawrin	2006	Germany	Caucasian	Glioma	HB	PCR-RFLP	94/25	39	51	4	129	59	10	13	2	33	17	0.428	6
Morimoto	2003	Japan	Asian	Sarcomas	NA	PCR-RFLP	143/102	54	72	17	180	106	39	50	13	128	76	0.624	10
Naidu	2009	Malaysia	Asian	Breast	HB	PCR-RFLP	387/252	179	172	36	530	244	132	105	15	369	135	0.322	9
Nan	2009	U.S.A.	Caucasian	Skin cancer	PB	TaqMan	768/833	365	325	78	1055	481	406	343	84	1155	511	0.359	11
Shen	2013	China	Asian	Gastric cancer	PB	Sequencing	304/392	118	124	62	360	248	132	188	72	452	332	0.724	11
Sheu	2015	China	Asian	HCC	HB	TaqMan	289/595	82	150	57	314	264	159	314	122	632	558	0.146	8
Spinola	2005	Italy	Caucasian	Lung	HB	Pyrosequencing	274/401	148	104	22	400	148	193	168	40	554	248	0.699	9
Spinola	2005	Italy	Caucasian	Breast	HB	Pyrosequencing	142/220	67	55	20	189	95	112	83	25	307	133	0.117	8
Spinola	2005	Italy	Caucasian	CRC	HB	Pyrosequencing	179/220	98	63	18	259	99	112	83	25	307	133	0.117	8
Tanuma	2010	Japan	Asian	OSCC	HB	PCR-SSCP	150/100	69	53	28	191	109	42	48	10	132	68	0.487	8
Tsay	2020	Taiwan	Asian	Cervical cancer	HB	TaqMan	428/856	114	222	92	450	406	242	426	188	910	802	0.984	10
Ture	2015	Turkey	Asian	Lung cancer	HB	PCR-RFLP	124/100	66	47	11	179	69	48	46	6	142	58	0.242	7
Wang	2004	U.S.A.	Caucasian	Prostate	PB	PCR-RFLP	284/97	125	117	42	367	201	53	40	4	146	48	0.291	8
Wang	2004	U.S.A.	African	Prostate	PB	PCR-RFLP	45/94	37	6	2	80	10	76	18	0	170	18	0.305	7
Whittle	2016	U.S.A.	Caucasian	Neuroblastoma	NA	PCR-RFLP	126/114	45	69	12	159	93	50	60	4	160	68	0.006	9
Wimmer	2019	Germany	Caucasian	HNSCC	PB	PCR-RFLP	284/123	188	84	12	460	108	55	60	8	170	76	0.114	9
Yang	2012	China	Asian	HCC	HB	TaqMan	711/740	216	351	144	783	639	247	361	132	855	625	0.996	10

First author	Year	Country	Ethnicity	Type of Cancer	Source of control	Genotyping method	Case/control	Cases					Controls					HWE	Score
Val^10^Ile rs1966265								CC	CT	TT	C	T	CC	CT	TT	C	T		
Chen	2018	Taiwan	Asian	Uterine Cervical	HB	TaqMan	227/335	61	105	61	227	227	91	168	76	350	320	0.927	9
Chou	2017	Taiwan	Asian	OSCC	PB	TaqMan	955/1191	213	514	228	940	970	285	580	326	1150	1232	0.391	11
FitzGerald	2009	U.S.A.	Caucasian	Prostate	PB	SNPlex	1259/1254	782	405	72	1969	549	742	447	65	1931	577	0.827	15
FitzGerald	2009	U.S.A.	African	Prostate	PB	SNPlex	147/80	132	15	0	279	15	70	10	0	150	10	0.551	13
Jiang	2015	China	Asian	Breast	NA	Snapshot	747/716	171	408	168	750	744	126	364	226	616	816	0.322	12
Nan	2009	U.S.A.	Caucasian	Skin cancer	PB	TaqMan	753/821	461	251	41	1173	333	507	271	43	1285	357	0.390	11
Sheu	2015	China	Asian	HCC	HB	TaqMan	289/595	65	160	64	290	288	151	300	144	602	588	0.835	8
Tsay	2020	Taiwan	Asian	Cervical cancer	HB	TaqMan	428/856	95	226	107	416	440	215	420	221	850	862	0.585	10

First author	Year	Country	Ethnicity	Type of cancer	Source of control	Genotyping method	Case/control	Cases					Controls					HWE	Score
rs7708357								GG	GA	AA	G	A	GG	GA	AA	G	A		
Chen	2018	Taiwan	Asian	Uterine cervical	HB	TaqMan	227/335	222	4	1	448	6	321	13	1	655	15	0.038	9
Chou	2017	Taiwan	Asian	OSCC	PB	TaqMan	955/1191	932	22	1	1886	24	1167	23	1	2357	25	0.015	11
FitzGerald	2009	U.S.A.	Caucasian	Prostate	PB	SNPlex	1258/1254	459	632	167	1550	966	507	569	178	1583	925	0.368	15
FitzGerald	2009	U.S.A.	African	Prostate	PB	SNPlex	146/78	49	74	23	172	120	34	35	9	103	53	0.999	13
Sheu	2015	China	Asian	HCC	HB	TaqMan	289/595	283	5	1	571	7	577	18	0	1172	18	0.708	8
Tsay	2020	Taiwan	Asian	Cervical cancer	HB	TaqMan	428/856	416	10	2	842	14	838	17	1	1693	19	0.005	9

First author	Year	Country	Ethnicity	Type of cancer	Source of control	Genotyping method	Case/control	Cases					Controls					HWE	Score
rs2011077								CC	CT	TT	C	T	CC	CT	TT	C	T		
Chen	2018	Taiwan	Asian	UT-cervical	HB	TaqMan	227/335	63	102	62	228	226	94	163	78	351	319	0.652	9
Chou	2017	Taiwan	Asian	OSCC	PB	TaqMan	955/1191	210	509	236	929	981	288	577	326	1153	1229	0.299	11
Ma	2008	Japan	Asian	Prostate	HB	PCR-RFLP	492/179	94	285	113	473	511	11	85	83	107	251	0.075	10
Sheu	2015	China	Asian	HCC	HB	TaqMan	289/595	66	159	64	291	287	147	297	151	591	599	0.968	8
Tsay	2020	Taiwan	Asian	Cervical cancer	HB	TaqMan	428/856	94	224	110	412	444	217	418	221	852	860	0.495	10

First author	Year	Country	Ethnicity	Type of cancer	Source of control	Genotyping method	Case/control	Cases					Controls					HWE	Score
rs376618								AA	AG	GG	A	G	AA	AG	GG	A	G		
FitzGerald	2009	U.S.A.	Caucasian	Prostate	PB	SNPlex	1238/1245	703	448	87	1854	622	712	437	96	1861	629	0.013	15
FitzGerald	2009	U.S.A.	African	Prostate	PB	SNPlex	145/80	65	59	21	189	101	38	38	4	114	46	0.154	13
Nan	2009	U.S.A.	Caucasian	Skin cancer	PB	TaqMan	762/830	451	273	38	1175	349	468	326	36	1262	398	0.026	11

### Quality assessment

Two investigators evaluated the quality of each study using the quality assessment criteria [52]. Quality scores of studies ranged from 0 (lowest) to 15 (highest). Studies with scores ≤9 were considered as low quality, while those with scores > 9 were considered as high quality.

### Statistical analysis

Meta-analysis was carried out using STATA 14.0 software (Stata Corporation, College Station, TX, U.S.A.). The Hardy–Weinberg equilibrium (HWE) of control genotypes was determined by the chi-square test.

The strength of the association between *FGFR4* polymorphisms and cancer susceptibility was evaluated by pooled odds ratios (ORs) and their 95% confidence intervals (CIs) in five (heterozygous, homozygous, dominant, recessive, and allele) genetic models. The significance of the pooled OR was assessed by the Z-test, and *P*<0.05 was considered to be statistically significant. The between-study heterogeneity was evaluated by the Q statistic. When the PQ < 0.1, indicating the presence of heterogeneity, the random-effects model was selected, otherwise, the fixed-effects model was chosen.

Publication bias was inspected by using Begg’s funnel plots and the asymmetric plots implied potential publication bias. Egger’s test was used to measure the degree of asymmetry. A *P*<0.05 indicated significant publication bias.

Sensitivity analyses was done to evaluate whether a single study influenced the overall pooled results by omitting each study in turn.

## Results

### Study characteristics

A total of 57 case–control studies from 30 published articles [13–42] that met the inclusion criteria were included in our meta‐analyses. Of these 57 studies, the FGFR4 rs351855 in 35 studies, rs1966265 in 8 studies, rs7708357 in 6 studies, rs2011077 in 5 studies, and rs376618 in 3 studies were analyzed, respectively. The characteristics and relevant data of the included studies are presented in Table 1.

### Meta-analysis results

**Figure 2:**
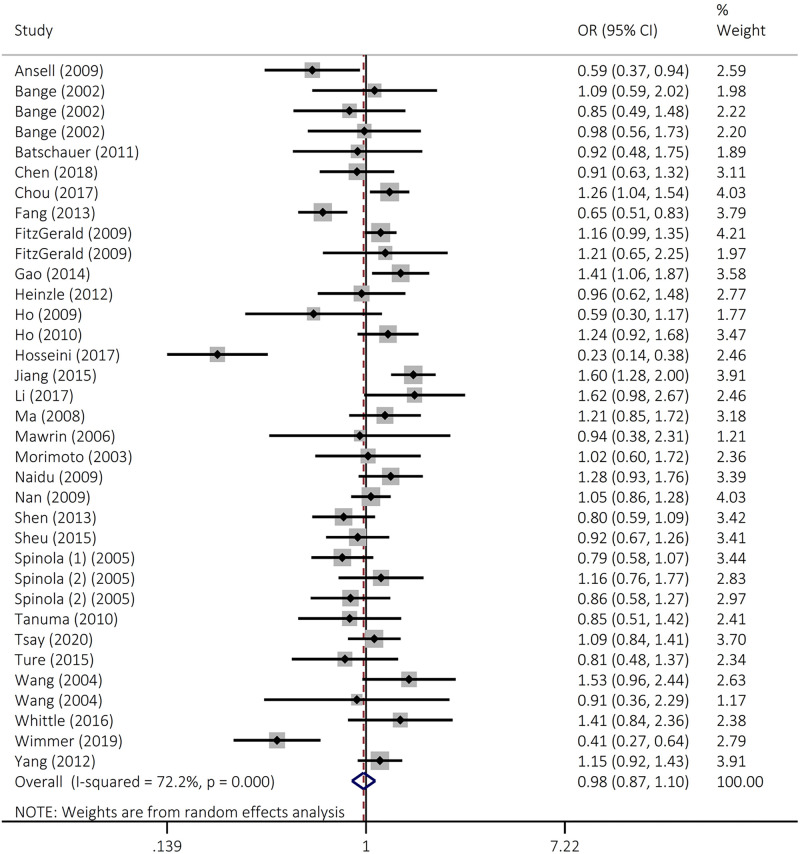
Forest plot for the association of the FGFR4 rs351855 polymorphism with overall cancer susceptibility in codominant (AG+AA vs GG)

**Figure 3:**
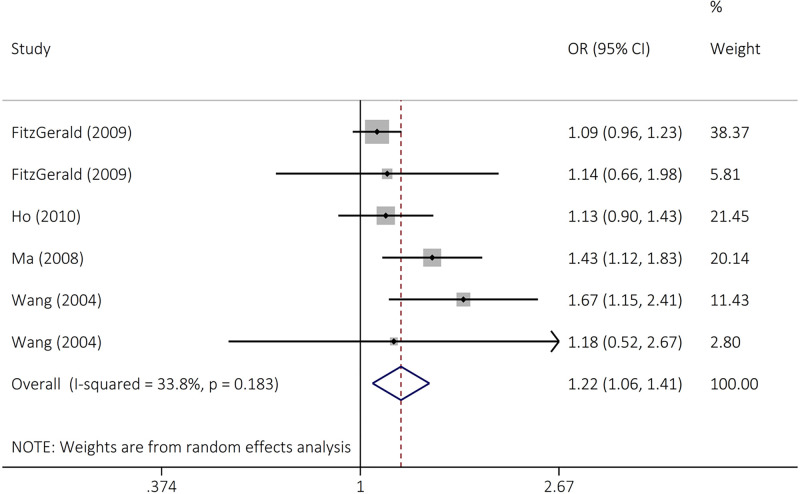
Forest plot for the association of the FGFR4 rs351855 polymorphism with prostate cancer susceptibility (A vs G)

The findings did not support an association between *FGFR4* rs351855 polymorphism and overall cancer susceptibility in heterozygous (OR = 0.97, 95% CI = 0.87–1.07, *P*=0.514, AG vs GG), homozygous (OR = 1.14, 95% CI = 0.95–1.37, *P*=0.166, AA vs GG), dominant (OR = 0.98, 95% CI = 0.87–1.10, *P*=0.686, AG+AA vs GG), recessive (OR = 1.15, 95% CI = 0.98–1.33, *P*=0.79, AA vs AG+GG), and allele (OR = 1.02, 95% CI = 0.93–1.12, *P*=0.663, A vs G) genetic models (Figure 2 and Table 2). Stratified analysis was achieved by ethnicity and cancer type (Table 3 and Figure 3). The results indicated that rs351855 variant significantly increased the risk of prostate cancer in heterozygous (OR = 1.16, 95% CI = 1.02–1.32, *P*=0.025 AG vs GG), dominant (OR = 1.20, 95% CI = 1.06–1.35, *P*=0.004, AG+AA vs GG), and allele (OR = 1.22, 95% CI = 1.06–1.41, *P*=0.005, A vs G) genetic models.

**Table 2:** The pooled ORs and 95% CIs for the association between FGFR4 polymorphisms and cancer susceptibility

	*n*	Genetic model	Association test			Heterogeneity test			Egger’s test *P*	Begg’s test *P*
			OR (95% CI)	Z	*P*	χ^2^	*I^2^* (%)	*P*		
Overall										
rs351855 G>A	35	AG vs GG	0.97 (0.87–1.07)	0.65	0.514	82.87	60.2	<0.0001	0.012	0.064
		AA vs GG	1.14 (0.95–1.37)	1.39	0.166	116.25	71.6	<0.0001	0.966	0.975
		AG+AA vs GG	0.98 (0.87–1.10)	0.40	0.686	122.33	72.2	<0.0001	0.061	0.129
		AA vs AG+GG	1.15 (0.98–1.33)	1.76	0.79	94.88	65.2	<0.0001	0.476	0.306
		A vs G	1.02 (0.93–1.12)	0.47	0.639	150.59	78.1	<0.0001	0.416	0.293
rs1966265 C>T	8	CT vs CC	1.01 (0.89–1.14)	0.14	0.891	11.12	37.1	0.133	0.739	1.000
		TT vs CC	0.94 (0.77–1.16)	0.56	0.574	14.60	58.9	0.024	0.373	0.176
		CT+TT vs CC	0.98 (0.87–1.11)	0.31	0.759	11.52	39.2	0.118	0.810	0.805
		TT vs CT+CC	0.87 (0.78–0.97)	2.61	**0.009**	14.07	57.3	0.029	0.094	0.051
		T vs C	0.95 (0.87–1.04)	1.03	0.303	14.24	50.8	0.047	0.722	0.805
rs7708357 G>A	6	AG vs GG	1.17 (0.95–1.44)	1.45	0.146	5.61	10.9	0.346	0.221	0.039
		AA vs GG	1.10 (0.87–1.40)	0.83	0.406	3.61	0.0	0.607	0.143	1.000
		AG+AA vs GG	1.17 (1.02–1.36)	2.19	**0.028**	4.72	0.0	0.451	0.467	0.091
		AA vs AG+GG	0.98 (0.79–1.21)	0.20	0.840	3.77	0.0	0.583	0.097	0.624
		A vs G	1.08 (0.98–1.20)	1.51	0.132	4.22	0.0	0.518	0.964	0.348
rs2011077 C>T	5	CT vs CC	1.03 (0.79–1.33)	0.21	0.831	11.30	64.6	0.023	0.054	0.014
		TT vs CC	0.79 (0.49–1.25)	1.02	0.309	28.54	86.0	<0.0001	0.228	0.327
		CT+TT vs CC	0.94 (0.69–1.28)	0.39	0.695	17.85	77.6	0.001	0.091	0.050
		TT vs CT+CC	0.79 (0.56–1.13)	1.27	0.203	28.84	86.1	<0.0001	0.681	1.000
		T vs C	0.89 (0.70–1.13)	0.97	0.332	33.89	88.2	<0.0001	0.380	0.327
rs376618 A>G	3	AG vs AA	0.95 (0.85–1.09)	0.56	0.753	1.76	0.0	0.414	0.761	0.602
		GG vs AA	1.04 (0.81–1.33)	0.29	0.771	4.12	51.5	0127	0.067	0.117
		AG+GG vs AA	0.97 (0.76–1.10)	0.45	0.654	1.27	0.0	0.531	0.858	0.602
		GG vs AG+AA	1.19 (0.74–1.93)	0.71	0.476	5.04	60.3	0.080	0.014	0.117
		G vs A	0.99 (0.90–1.09)	0.20	0.841	2.21	9.5	0.331	0.383	0.602

**Table 3:** Stratified analysis of rs351855 polymorphisms by ethnicity and cancer type

	*n*	Genetic model	Association test			Heterogeneity test			Egger’s test *P*	Begg’s test *P*
			OR (95% CI)	Z	*P*	χ^2^	*I^2^* (%)	*P*		
Caucasian	15	AG vs GG	0.97 (0.84–1.12)	0.42	0.672	26.56	47.3	0.002	0.162	0.586
		AA vs GG	1.11 (0.95–1.29)	1.30	0.193	19.36	27.7	0.152	0.331	0.216
		AG+AA vs GG	0.96 (0.83–1.12)	0.47	0.636	35.15	57.3	0.002	0.257	0.471
		AA vs AG+GG	1.09 (0.94–1.26)	1.09	0.278	16.49	15.1	0.284	0.118	0.125
		A vs G	1.03 (0.92–1.15)	0.43	0.666	30.34	53.9	0.007	0.789	0.458
Asian	17	AG vs GG	0.96 (0.82–1.12)	0.56	0.572	55.29	71.1	0.000	0.023	0.039
		AA vs GG	1.1 (0.84–1.44)	0.72	0.470	94.65	83.1	0.000	0.636	0.510
		AG+AA vs GG	0.98 (0.81–1.17)	0.27	0.786	86.32	81.5	0.000	0.092	0.070
		AA vs AG+GG	1.13 (0.91–1.40)	1.12	0.262	76.08	79.0	0.000	0.832	0.458
		A vs G	1.01 (0.87–1.16)	0.11	0.913	119.83	86.6	0.000	0.352	0.217
Breast cancer	7	AG vs GG	0.94 (0.66–1.33)	0.35	0.729	26.00	76.9	0.000	0.050	0.099
		AA vs GG	1.03 (0.48–2.22)	0.08	0.939	44.25	86.4	0.000	0.358	0.186
		AG+AA vs GG	0.91 (0.58–1.44)	0.40	0.691	50.88	88.2	0.000	0.135	0.099
		AA vs AG+GG	1.05 (0.56–1.96)	0.16	0.877	31.50	81.0	0.000	0.540	0.176
		A vs G	0.92 (0.61–1.38)	0.40	0.690	71.30	91.6	0.000	0.233	0.072
Prostate cancer	6	AG vs GG	1.16 (1.02–1.32)	2.25	**0.025**	2.91	0.0	0.714	0.422	0.188
		AA vs GG	1.60 (0.98–2.61)	1.90	0.058	13.39	62.7	0.020	0.378	0.462
		AG+AA vs GG	1.20 (1.06–1.35)	2.89	**0.004**	1.67	0.0	0.892	0.639	0.851
		AA vs AG+GG	1.56 (0.92–2.65)	1.63	0.103	17.29	71.1	0.004	0.452	0.624
		A vs G	1.22 (1.06–1.41)	2.81	**0.005**	7.55	33.8	0.183	0.279	0.260
Gastrointestinal cancer	7	AG vs GG	0.92 (0.80–1.06)	1.17	0.243	6.73	10.9	0.346	0.071	0.090
		AA vs GG	1.06 (0.88–1.28)	0.63	0.528	3.72	0.0	0.715	0.581	0.881
		AG+AA vs GG	0.95 (0.84–1.09)	0.70	0.487	6.14	2.2	0.408	0.093	0.652
		AA vs AG+GG	1.10 (0.94–1.30)	1.17	0.241	2.34	0.0	0.886	0.824	0.762
		A vs G	1.01 (0.92–1.10)	0.16	0.873	4.37	0.0	0.627	0.172	0.230

Bold values denote statistical significance at the *P* <0.05 level.

**Figure 4:**
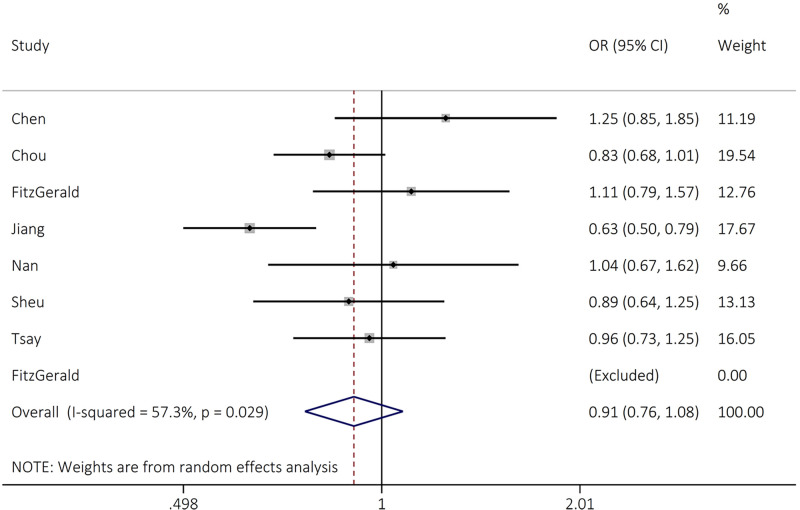
Forest plot for the association between FGFR4 rs1966265 and overall cancer risk in recessive (TT vs CT+CC) models

For *FGFR4* rs1966265 polymorphism, the findings revealed that this variant significantly reduced the risk of cancer susceptibility in recessive (OR = 0.87, 95% CI = 0.78–0.97, *P*=0.009, TT vs CT+CC) model (Table 2 and Figure 4).

**Figure 5:**
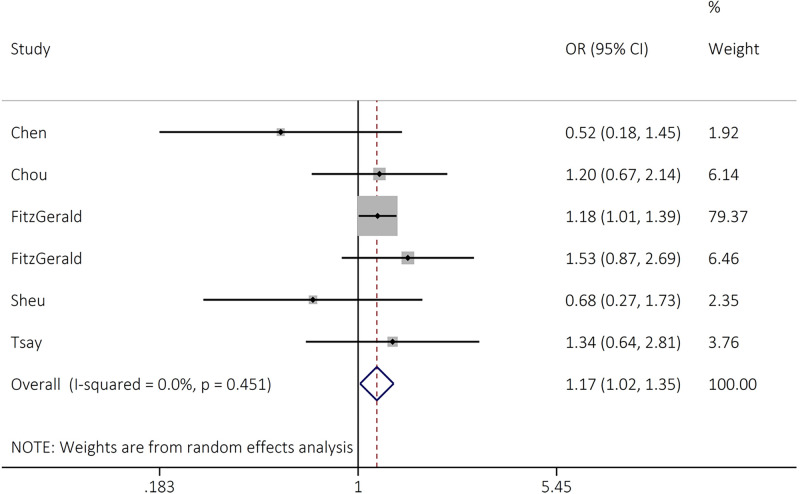
Forest plot for the association between FGFR4 rs7708357 and overall cancer risk in dominant model (AG+AA vs GG)

The rs7708357 variant of *FGFR4* significantly increased the risk of cancer development in dominant (OR = 1.17, 95% CI = 1.02–1.36, *P*=0.028, AG+AA GG) genetic model (Table 2 and Figure 5).

The rs2011077 and rs376618 variants were not associated with overall cancer risk in any genetic models tested (Table 2).

### Heterogeneity and publication bias

As shown in Table 2, heterogeneity among the studies was observed in all genetic comparisons for rs351855 and rs2011077. For rs1966265, heterogeneity was not found in heterozygous and dominant genetic models. While, heterogeneity was not detected in all genetic models for rs7708357 and rs376618.

**Figure 6:**
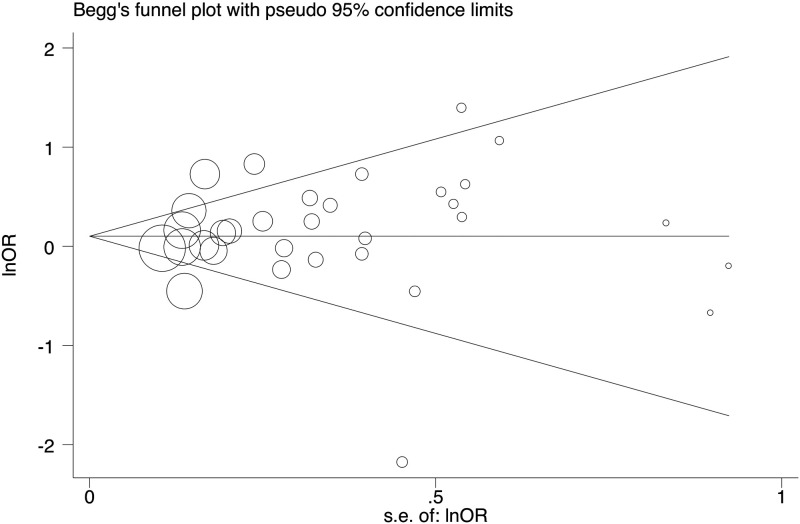
Begg’s funnel plot for the test of publication bias for FGFR4 rs351855 in recessive model (AA vs AG+GG)

The potential publication bias was evaluated using Begg’s funnel plot and Egger’s test. The shape of funnel plots was symmetrical and the Egger’s test supported no existence of publication bias in all comparison except rs351855 polymorphism in heterozygous and rs376618 polymorphism in recessive genetic model (Table 2 and Figure 6).

### Sensitivity analysis

**Figure 7:**
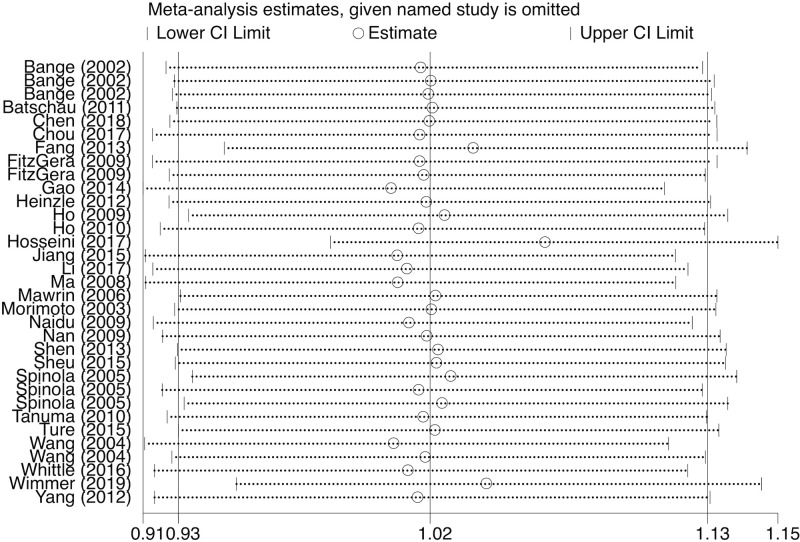
Sensitivity analysis on the association between the rs351855 polymorphism and susceptibility of overall cancer in allele genetic model (A vs G)

We performed sensitivity analysis to assess the effect of a specific publication on the overall estimate. For rs351855, the pooled ORs showed no significant change appeared when each study was neglected, one at a time, in heterozygous, dominant, and allele genetic models (Figure 7). For rs1966265, sensitivity analysis indicated no changes of results in heterozygous, homozygous, dominant, recessive, and allele genetic models. For rs7708357, no alterations of results were detected in homozygous, recessive, and allele genetic models. Thus, the final pooled results are both stable and reliable.

## Discussion

FGFs and their receptors (FGFRs) regulate numerous cellular processes including the regulation of cell proliferation, differentiation, migration, and metabolism [12]. Deregulation of FGFRs signaling have been found to play an important role in cancer development and progression as well as resistance to anticancer [53–55]. Overexpression of FGFR4 predict metastasis and poor survival outcome in various cancers [56–58]. Blocking FGFR4 significantly suppresses the cancer and indicates that FGFR4 is a potential target for the cancer treatment [59]. Polymorphisms in the FGFR4 rs351855 (Gly^388^Arg) polymorphism, is positioned in the transmembrane domain of the EGFR4. It has been found that Arg^388^ allele causes increased receptor stability and prolonged receptor activation [60].

Several reports have examined the relationship between FGFR4 gene polymorphisms and diverse cancer types [13–20,22–42]. However, the findings were inconsistent. Therefore, this updated meta-analysis including more eligible studies was performed to evaluate the impact of *FGFR4* polymorphisms on cancer susceptibility. For *FGFR4* rs351855 polymorphism, the findings from 34 studies including 10407 cases and 12382 controls did not support an association between this polymorphism and overall cancer susceptibility. Stratified analyses showed that this SNP significantly increased the risk of prostate cancer (*n*=6) in heterozygous, homozygous, dominant, and allele genetic models. The variant was not related to breast cancer as well as gastrointestinal cancer. Furthermore, the variant was not correlated with ethnicity. A meta-analysis performed by Xiong et al. [51] from 27 studies indicated a significant association between *FGFR4* rs351855 polymorphism and overall cancer risk in recessive genetic model. Stratified analysis showed that rs351855 SNP significantly increased the risk of prostate cancer. A meta-analysis performed by Shu et al. [61] on 14 studies investigated the association between *FGFR4* rs351855 polymorphism and various cancer risks indicated a significant association between this SNP and risk of overall cancer in all heterozygous, homozygous, dominant, recessive, and allele tested genetic models.

*FGFR4* rs1966265 changes chemotherapy response in breast cancer [62], higher risk of oral squamous cell carcinoma susceptibility [31], initiation of cervical cancer (Taiwanese women) [19], and higher risk of breast cancer in Chinese women of Heilongjiang province [16]. *FGFR4* rs2011077 TC+CC polymorphism is associated with higher tumor stage, tumor size, and grading in urothelial cell carcinoma [21]. *FGFR4* rs2011077 with the GG genotype also increased the risk of prostate cancer in Japanese population [26].

To the best of our knowledge, for the first time, we performed pooled analysis to inspect the impact of rs1966265, rs7708357, rs2011077, and rs376618 polymorphisms and overall cancer risk.

For *FGFR4* rs1966265 polymorphism, the findings revealed that this variant significantly reduced the risk of cancer susceptibility in recessive (OR = 0.87, 95% CI = 0.78–0.97, *P*=0.009, TT vs CT+CC) model (Table 2 and Figure 3). Regarding rs7708357 polymorphism, the finding indicated that the rs1966265 variant significantly increased the risk of overall cancer in dominant (OR = 1.17, 95% CI = 1.02–1.36, *P*=0.028, AG+AA GG) genetic model (Table 2 and Figure 4). While, the rs2011077 and rs376618 polymorphisms were not associated with cancer risk in any genetic models tested (Table 2).

Some limitations of this meta-analysis should be taken into account. First, the sample sizes of this meta-analysis were not large especially for rs1966265 (*n*=7 studies), rs7708357 (*n*=5 studies), rs2011077 (*n*=4 studies), and rs376618 (*n*=3 studies) polymorphisms as well as in stratified analyses, which may lead to reduced statistical power. Second, the strength of the association were measured by unadjusted ORs for confounding factors due to the lack of demographic and environmental factors, which might have affected the results. Third, publication bias may be unavoidable since we were only able to acquire data from published articles. Finally, the meta-analysis was associated with a significant heterogeneity in some polymorphisms.

The current investigation provided a source for basic medical scientist and clinician to understand the importance of FGFR4 in different types of cancers and use the results as potential biomarkers for susceptibility to cancers. It also provided a collection of previous investigation on this gene to help epidemiologist scientists for their future investigations (Rev 1-4).

In summary, this meta-analysis revealed that FGFR4 rs351855 (Gly^388^Arg) polymorphism might be a marker for susceptibility to prostate cancer. The rs1966265 polymorphism significantly decreased and rs1966265 polymorphism significantly increased the risk of overall cancer. No significant associations were found for the *FGFR4* rs2011077 and rs376618 polymorphisms. However, these findings need to be further confirmed through large samples and different ethnic populations.

## Abbreviations

CD334: cluster of differentiation 334
CI: confidence interval
EGFR: epidermal growth factor receptor
EMT: epithelial-to-mesenchymal transition
FGF: fibroblast growth factor
FGFR: FGF receptor
HNSCC: head and neck squamous cell carcinoma
MAPK: mitogen-activated protein kinase
OR: odds ratio
PI3K: Phosphoinositide 3-kinases
PLCγ: phospholipase C gamma
RTK: receptor tyrosine kinases
SNP: single nucleotide polymorphism
STAT: signal transducer and activator of transcription
